# Determination of Bioactive Components and Antimicrobial Activity of Bee Pollen and Investigation of Food Safety Hazards in Terms of Microplastics-Related Chemical Markers

**DOI:** 10.3390/foods15122115

**Published:** 2026-06-12

**Authors:** Selçuk Alan, Gönül Damla Büyük, Mehmet Emin Aydemir

**Affiliations:** 1 Department of Food Hygiene and Production, Faculty of Veterinary Medicine, Kafkas University, 36000 Kars, Türkiye; 2 Department of Food Hygiene and Technology, Faculty of Veterinary Medicine, Harran University, 63290 Şanlıurfa, Türkiye

**Keywords:** bee pollen, phenolic compounds, antimicrobial activity, PY-GC/MS, microplastic-related markers

## Abstract

This study evaluated the microbiological quality, phenolic compound profile, antimicrobial activity against foodborne pathogens, and the presence of potential chemical markers associated with microplastic polymers in 35 commercial bee pollen samples obtained from the seven geographical regions of Türkiye. Microbiological analyses included the enumeration of total mesophilic aerobic bacteria, coliforms, yeasts and molds, lactobacilli, lactococci, and psychrophilic bacteria. Antimicrobial activity was determined against *Escherichia coli* O157:H7, *Staphylococcus aureus*, and *Salmonella Enteritidis* using the disk diffusion method. Phenolic compounds were analyzed by HPLC-DAD, while characteristic pyrolysis products associated with microplastics were analyzed by PY-GC/MS. The results indicated that the pollen samples generally exhibited low microbial contamination levels and variable antimicrobial activity, depending on their geographical origin. Quercetin was identified as the predominant phenolic compound, and samples with higher phenolic content tended to show stronger antimicrobial effects, particularly against *S. aureus*. PY-GC/MS analyses revealed the presence of several chemical markers potentially associated with plastic polymers in a considerable proportion of the samples. Spearman correlation analysis showed a positive correlation between total phenolic content and particularly *S. aureus* inhibition. These findings highlight the nutritional and functional value of bee pollen while also drawing attention to emerging food safety concerns related to possible exposure to plastic-associated environmental contaminants. Regular monitoring of bee pollen is therefore recommended to ensure product quality and consumer safety.

## 1. Introduction

Bee pollen is a highly nutritious natural bee product formed when worker bees collect pollen from flowers, mix it with plant nectar and salivary secretions, and deposit it as pellets at hive entrances [1,2]. Containing carbohydrates (13–55%), protein (10–40%), essential amino acids, lipids, dietary fiber, vitamins, and minerals, bee pollen is regarded as a nutritionally valuable natural product [1,2,3]. In addition to essential nutrients, bee pollen is extremely rich in bioactive components such as phenolic acids (chlorogenic, caffeic, p-coumaric acid, etc.) and flavonoids (rutin, quercetin, kaempferol, etc.) that confer its functional properties [4,5,6]. These secondary metabolites are associated with antioxidant, antimicrobial, antifungal, and anti-inflammatory properties, including inhibitory effects against several foodborne pathogens [7,8]. Despite these valuable nutritional and functional properties of pollen, the risk of contamination with environmental pollutants should not be ignored in terms of food safety. In particular, microplastics, which have become widespread in the environment in recent years, are attracting attention as a potential contaminant in many food matrices, including natural products. With industrialization and the rapid increase in plastic production, microplastics, defined as particles <5 mm in size, have become widespread enviromental pollutants that threaten air, water, soil and other environmental compartments [9]. Currently, dietary exposure to microplastics through food and beverages is considered an emerging public health concern, as these particles may accumulate in the human body, including in blood, tissues, and organs, and potentially disrupt cellular functions [10].

Honeybees (*Apis mellifera*), with foraging ranges that may extend up to 10 km, can act as environmental bioindicators by collecting particles from their surroundings [11,12]. During their flights and foraging activities, bees collect microplastics from the air, polluted water sources, or particles on the flowers they visit and carry them to the hive [9,10]. In addition, various beekeeping practices, such as plastic frames used in hive management, synthetic cover cloths, or microfibers shed from beekeeping clothing, may contribute to the presence of these plastic-related chemical markers [9,11]. Recent studies have increasingly indicated that honeybees and bee products may serve as useful matrices for monitoring environmental microplastic pollution. Gómez-Méndez et al. [13] reported the presence of microplastics in *Apis mellifera* bees, pollen, and honey collected from both urban and rural areas, with fibers being the most frequently observed particle type. Similarly, Cortés-Corrales et al. [14] evaluated honeybees, pollen, and an in-hive passive sampler as matrices for monitoring airborne microplastics and detected microplastics in pollen samples, identifying polymers such as polyethylene terephthalate (PET), polypropylene (PP), polyethylene (PE), polyacrylonitrile (PAN), and polyamide (PA). In addition, Schiano et al. [15] demonstrated the occurrence of microplastics and microfibers in honeybees and beehive products and suggested that contamination may originate from several pathways, including airborne deposition, hive components, flowers, interactions among nestmates, beekeeping clothing, and surrounding agricultural or urban environments.

Despite these findings, knowledge regarding microplastic-related contamination in bee pollen remains limited compared with other food matrices. Moreover, most previous studies have focused mainly on visual or spectroscopic identification of particles, whereas data based on pyrolysis-derived chemical markers in commercial ready-to-eat bee pollen are still scarce. Studies simultaneously evaluating microbiological quality, phenolic composition, antimicrobial activity, and potential microplastic-related chemical markers of commercial bee pollen also remain limited in the literature. Therefore, investigating potential polymer-related pyrolysis markers in bee pollen, together with its microbiological quality, phenolic profile, and antimicrobial activity, may provide a broader food safety perspective on this increasingly consumed natural product. In this context, this study aimed to provide a holistic food safety assessment of commercial bee pollen samples collected from different geographical regions. Specifically, the objectives were: (i) to determine the microbiological quality of the samples; (ii) to characterize their phenolic compound profiles; (iii) to evaluate their antimicrobial activity against important foodborne pathogens, including *E. coli* O157:H7, *S. Enteritidis*, and *S. aureus*; (iv) to investigate the presence and concentration levels of possible chemical markers potentially associated with microplastic polymers.

## 2. Materials and Methods

### 2.1. Materials

**Figure 1:**
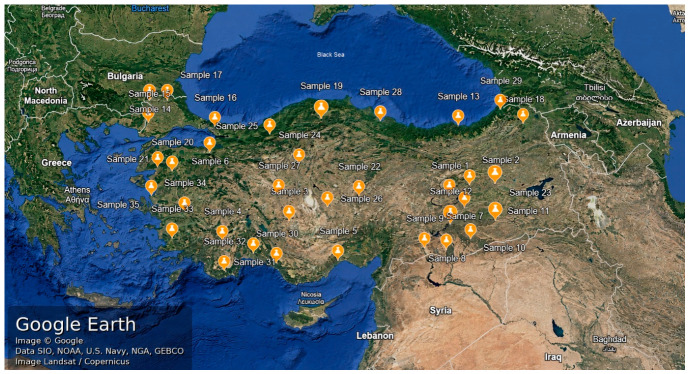
Map of the study area showing the locations of the collected pollen samples. Base map source: Google Earth; Image © Google; Data SIO, NOAA, U.S. Navy, NGA, GEBCO; Image Landsat/Copernicus. Map lines delineate study areas and do not necessarily depict accepted national boundaries.

In this study, a total of 35 commercial bee pollen samples (five samples from each of Türkiye’s seven geographical regions) were collected to provide a broad geographical overview of commercial bee pollen marketed in Türkiye. A formal randomization procedure was not applied; instead, a regional stratified sampling approach was used to ensure representation of all geographical regions of Türkiye. The sampling locations were visualized using Google Earth Pro software, version 7.3 (Google LLC, Mountain View, CA, USA) to demonstrate the spatial distribution of the study area (Figure 1). Pollen samples sold as ready-to-eat food were purchased from retail outlets such as supermarkets and local food shops with their original packaging intact (each sample weighing at least 100 g). The samples were from the 2025 production season and were selected from products released to the market following the harvest period. During sampling, the production and expiration dates of the products were recorded, and products with different packaging types (glass jars, plastic containers, etc.) were specifically noted. This information was considered for the evaluation of possible plastic-related contamination. The collected samples were brought to the laboratories of the Department of Food Hygiene and Production, Faculty of Veterinary Medicine, Kafkas University for microbiological and chemical analyses and were stored under appropriate conditions (refrigerated and protected from light) until the analysis date.

### 2.2. Methods

#### 2.2.1. Microbiological Quality Analyses

To determine the general microbial quality of pollen samples, 25 g of each pollen sample was weighed under aseptic conditions, placed in sterile sample bags (Seward, Worthing, UK), and homogenized in a stomacher device (Bagmixer^®^, Interscience, Saint-Nom-la-Bretèche, France) after adding 225 mL of 0.1% Peptone Water (Merck, Darmstadt, Germany). Appropriate serial dilutions were prepared from the resulting homogenates and inoculated onto specific culture media. For total mesophilic aerobic bacteria count, Plate Count Agar (PCA) (Neogen Corporation NCM0010A, 620 Lesher Place, Lansing, MI, USA) was used at 37 °C for 24–48 h [16]; for total psychrophilic aerobic bacteria count, PCA was used at 4 °C for 7–10 days; for coliform bacteria, incubation was performed on Violet Red Bile Agar (VRB) (Biokar Diagnostics BK152HA, Rue des 40 Mines, Allonne, France) at 37 °C for 24 h [17]; for enumeration of yeasts and molds, incubation was performed on Potato Dextrose Agar (PDA) (Oxoid Ltd., Basingstoke, UK) at 25 °C for 5 days. In addition, for enumeration of lactobacilli, Man Rogosa Sharpe Agar (MRS) (Chemsolute, 8761.0500, Th. Geyer GmbH & Co. KG, Renningen, Germany) was incubated at 30 °C for 72 h, and for enumeration of lactococci, M17 Agar (M17, Oxoid Ltd., Basingstoke, UK) was incubated at 30 °C for 48 h, after which specific colonies were counted.

#### 2.2.2. Determination of Antimicrobial Activity

The antimicrobial activity of bee pollen samples against foodborne pathogens was evaluated using the disc diffusion test. The reference strains *E. coli* O157:H7 (ATCC 43895), *S. Enteritidis* (NCTC 12694), and *S. aureus* (ATCC 25923) used in the study were activated on PCA medium and incubated at 37 °C for 24 h in an aerobic environment. Bacterial suspensions were prepared from the developing fresh colonies in 5 mL of physiological saline (FS), and their turbidity was adjusted to the McFarland 0.5 standard (approximately 1.5 × 10^8^ cfu/mL). Suspensions were prepared from each pollen sample and sterilized by filtration. For the test, Mueller–Hinton agar media were poured into 90 mm sterile Petri dishes to a depth of 4.0 ± 0.5 mm, and 100 µL of the prepared standard bacterial suspensions were taken and inoculated using the spread plate technique [7]. Pollen samples were suspended in sterile distilled water at a concentration of 100 mg/mL and homogenized by vortexing. Approximately 10 µL of the resulting suspensions were taken and loaded onto sterile blank discs, with each disc containing approximately 1 mg of crude pollen equivalent. Blank discs were placed on the agar and incubated at 37 °C for 18–24 h. The disk diffusion assay was selected as a rapid screening method to compare the antimicrobial potential of different bee pollen samples. Determination of MIC and MBC values and the use of purified pollen extracts were beyond the scope of the present study. Standard antibiotic discs containing gentamicin and ampicillin (Bioanalyse CN10) were used as positive controls. Sterile distilled water, used as the suspension medium for the pollen samples, was included as a negative control in the disc diffusion assay. After incubation, inhibition zone diameters were measured in millimeters (mm) and reported descriptively; standard antibiotic discs were used as positive controls. All analyses were performed in triplicate.

#### 2.2.3. Chemical Analyses

To determine the acidity of pollen samples, 5 g of pollen sample was diluted with 45 mL of distilled water, and pH measurements were performed using a digital pH meter (Orion Star A221, Thermo Fisher Scientific Inc., Waltham, MA, USA).

**Determination of Phenolic Compound Profile and Concentrations**: Analyses of specific phenolic acids and flavonoids in bee pollen samples were performed using an Agilent 1200 Infinity Series High-Performance Liquid Chromatography system equipped with a Diode Array Detector (HPLC-DAD; Agilent Technologies, Santa Clara, CA, USA) [1,18]. Homogenized pollen samples were mixed with a suitable extraction solvent. One gram of homogenized bee pollen was mixed with 10 mL of an 80% ethanol–20% water solution and extracted in an ultrasonic bath (WiseClean, Daihan Scientific Co., Ltd., Wonju, Republic of Korea) to break down the pollen exine (outer) wall and maximize the transfer of intracellular phenolics into the solvent. After centrifugation of the mixture, the resulting supernatant was filtered through syringe filters with a pore size of 0.45 µm to remove any possible particles before instrument injection and transferred to HPLC vials. During chromatographic separation, the column temperature was fixed at 30 °C, and 10 µL of each sample was injected into the instrument. The mobile phase system used was (A) ultrapure water containing 0.1% phosphoric acid and (C) 100% acetonitrile (ACN). The flow rate was set at 0.8 mL/min throughout the analysis, and a specific gradient elution program lasting 40 min was applied. Chromatographic analyses were performed using an ACE Generix 5 C18 column (250 × 4.6 mm i.d., 5 µm particle size; Advanced Chromatography Technologies Ltd., Aberdeen, UK). The gradient elution program was as follows: 0 min, 83% A/17% C; 7 min, 85% A/15% C; 20 min, 80% A/20% C; 24 min, 75% A/25% C; 28 min, 70% A/30% C; 30 min, 60% A/40% C; 32 min, 50% A/50% C; 36 min, 30% A/70% C; and 40 min, 83% A/17% C. Quantification of phenolic compounds was performed using external standard calibration curves prepared individually for each analyte. Depending on the compound, calibration ranges varied between 1.5 and 200 ng/µL, and linear calibration models were used for quantification. Detection of phenolic compounds and integration of peak areas were performed on a DAD at wavelengths of 200 and 300 nm (reference: 100 and 500 nm).

**Detection of Chemical Markers Potentially Associated with Microplastic Polymers:** The detection of characteristic pyrolysis products that can be associated with plastic polymers in bee pollen samples was performed using pyrolysis–gas chromatography/mass spectrometry (PY-GC/MS). The selected pyrolysis markers were chosen based on previous studies reporting their association with commonly detected plastic polymers in environmental and food-related microplastic research, including polyethylene (PE), polypropylene (PP), polyvinyl chloride (PVC), polyethylene terephthalate (PET), and polycarbonate (PC). The analyses were performed using a Shimadzu GCMS-QP2010 Ultra gas chromatography–mass spectrometry system (Shimadzu Corporation, Kyoto, Japan) connected to a One Shot Pyrolyzer system (Frontier Laboratories Ltd., Koriyama, Japan). The pyrolysis oven temperature was set to 550 °C, the interface temperature to 320 °C, and the samples were analyzed in Eco-cup reaction tubes. Helium was used as the carrier gas for chromatographic separation, and injection was performed in split mode at a split ratio of 1/20. The injector temperature was set to 300 °C, the column flow rate to 1 mL/min, and the total flow rate to 27 mL/min. An Rtx-5MS (30 m × 0.25 mm × 0.25 μm; Restek Corporation, Bellefonte, PA, USA) or TRB-5MS capillary column (30 m × 0.25 mm × 0.25 μm; Teknokroma Analítica S.A., Sant Cugat del Vallès, Spain) was used for separation. The oven temperature program was applied as follows: holding at 40 °C for 5 min, then increasing to 320 °C with an increment of 10 °C/min and holding at this temperature for 20 min. Under mass spectrometry conditions, the ionization energy was set to 70 eV, the ion source temperature to 230 °C, the scan rate to 0.3 s, and the scan range to m/z 30–500. For the identification of polymers, 1,13-tetradecadiene and 1-heneicosene/1-tetradecene were used for PE; 2,4-dimethyl-1-heptene and 2,4,6,8-tetramethyl-1-undecene for PP; naphthalene and benzene for PVC; benzophenone and ethane-1,2-diylbenzoate for PET; p(4)-isopropenylphenol and phenol for PC; dipentene and D-limonene for NR; and 4-vinyl-cyclohexene and 4-phenylcyclohexene for SBR [19,20,21,22,23]. In this context, characteristic pyrolyzates specific to polymers and their m/z ions were considered, the obtained peak areas were evaluated using GCMSsolution software, version 2.72 (Shimadzu Corporation, Kyoto, Japan), and the analysis results were interpreted.

#### 2.2.4. Statistical Analysis

Statistical evaluation of all microbiological, chemical, and instrumental (PY-GC/MS and HPLC-DAD) datasets obtained within the scope of the study was performed using the SPSS 26.0 software package (SPSS statistics software, version 26.0, IBM Corp., Armonk, NY, USA). Microbiological analyses and antimicrobial activity tests were performed in triplicate. The determination of phenolic compounds and microplastic-related chemical markers was performed as single instrumental measurements due to instrument time limitations, sample throughput considerations, and analytical costs. Therefore, the obtained values should be interpreted as indicative rather than absolute quantitative estimates. This represents a limitation of the present study and should be considered when evaluating the results. While evaluating the data, frequency distributions were calculated for categorical variables, and descriptive statistics (mean ± standard deviation) were computed for numerical variables. The normality of the data was assessed using the Shapiro–Wilk test. Since most variables did not follow a normal distribution (*p* < 0.05), non-parametric tests were applied. Therefore, Spearman correlation analysis was applied to evaluate the pairwise relationships between the variables.

## 3. Results

### 3.1. Microbiological Quality Results

The enumeration results (log CFU/g) to determine the overall microbial quality are presented in Table 1. The data obtained reveal that the microbial load of pollen samples varied markedly among regions. The Total Mesophilic Aerobic Bacteria (TMAB) count, an indicator of the overall contamination level, was found to be above the detectable limits in 27 out of 35 samples (77.1%). The TMAB count in samples showing growth was calculated as an average of 2.26 ± 0.44 log CFU/g, while the highest regional contamination was detected in samples from the Eastern Anatolia Region (especially in sample number 18 with 3.23 log CFU/g). In contrast, almost all samples collected from the Mediterranean Region exhibited the cleanest profile, remaining below the detection limit from a microbiological perspective. Cold-growing psychrophilic aerobic bacteria were not detected in any sample (<1.00 log CFU/g).

Coliform bacteria, an important indicator of food hygiene and sanitation conditions, were below the detection limit in the vast majority of samples (71.5%). In the 10 samples where growth was detected, the average level was as low as 1.43 ± 0.49 log CFU/g; however, relatively higher levels were detected regionally in the Eastern Anatolia Region (sample no. 18, 2.61 log CFU/g) and partly in the Black Sea Region samples compared to other regions. Yeasts and molds were the most frequently detected microbial group in the pollen samples (74.2%) and reached the highest levels in samples from the Eastern Anatolia and Aegean Regions, with the maximum value recorded in Eastern Anatolia (4.49 log CFU/g). Lactobacilli, beneficial microorganisms naturally present in the fermentation processes of bee pollen, were detected in 25.7% of the samples, while lactococci were detected in 48.5%. When examining the regional distribution of lactic acid bacteria (LAB), it was determined that samples from Eastern Anatolia and the Aegean Region (particularly samples 20 and 21) had the richest profile in terms of beneficial flora.

### 3.2. Antimicrobial Activity Results

The antimicrobial activity of bee pollen samples collected within the scope of the study against *E. coli* O157:H7, *S. aureus*, and *S. Enteritidis*—foodborne pathogens of concern—was evaluated using the disc diffusion test, and the information on the obtained inhibition zone diameters (mm) is presented in Table 2.

**Figure 2:**
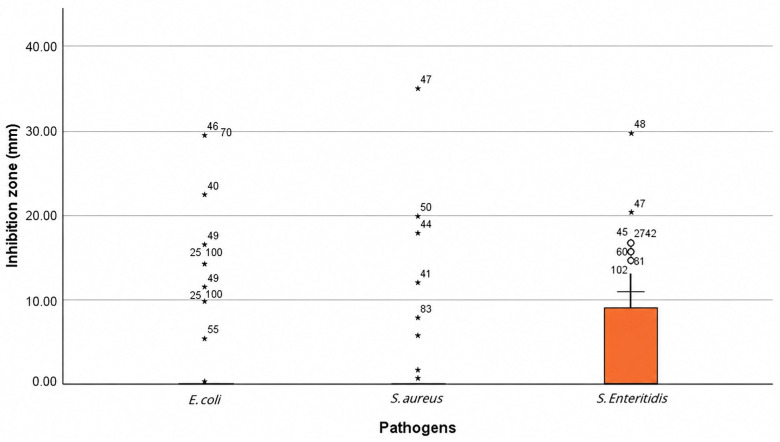
Distribution of antimicrobial activity against tested pathogens (inhibition zone, mm). Circles (dots) indicate outlier observations, whereas asterisks (*) indicate extreme outliers. Numbers shown next to the symbols represent the corresponding sample identification codes.

The findings revealed that the antimicrobial capacity of bee pollen samples was limited. While no inhibition zone (0 mm) was observed in the majority of the 35 samples examined (between 74.2% and 85.7% depending on the pathogen), relatively large inhibition zones were observed in the positive samples. The distribution of antimicrobial activity among the samples was visualized using a boxplot graph (Figure 2). The graph shows that the antimicrobial activity was not homogeneously distributed among the samples, and significantly high inhibition zones were observed in some samples.

A positive inhibition zone was obtained in 8 samples (22.8%) against the Gram-negative pathogen *E. coli* O157:H7 strain. The highest antimicrobial effect was observed in samples number 16 from the Marmara Region and number 24 from the Central Anatolia Region, with inhibition zones of 31 mm. The inhibition zone diameters observed in these two samples were found to be close to or larger than the standard gentamicin disc (29 mm) used as a positive control; however, these results should not be considered directly equivalent to antibiotic activity.

Although activity against Gram-positive *S. aureus* bacteria was observed in only 5 samples (14.2%), the average activity (20.40 ± 9.94 mm) was found to be high in these positive samples. In particular, sample number 16 from the Marmara Region formed a very large inhibition zone of 36 mm on *S. aureus*, which was determined to be higher than the gentamicin disc (27 mm); however, these results should not be equated directly with antibiotic activity.

Antimicrobial activity against *S. Enteritidis*, a common cause of foodborne infections, was determined in 9 samples (25.7%). The highest activity against this pathogen was observed in pollen sample number 16 (33 mm) from the Marmara Region. The diameter of this inhibition zone was observed to be higher than that of the gentamicin disc (26 mm) used as a positive control. However, this result should not be considered directly equivalent to antibiotic activity.

A general assessment at the regional level reveals that samples from the Marmara Region (particularly samples 14, 15, 16, and 17) exhibited simultaneous and high antimicrobial activity against all three tested pathogens. No inhibition zone was observed for the negative control (sterile distilled water) discs against any of the tested microorganisms. However, the findings indicate that the antimicrobial effect stems from a complex phenolic structure that cannot be reduced to a single component.

### 3.3. Phenolic Compound Profile of Bee Pollen Samples

In this study, High-Performance Liquid Chromatography (HPLC) analysis was performed to determine the phenolic profile and concentration of 35 bee pollen samples collected from seven different geographical regions. Eighteen different phenolic acids and flavonoids were screened in the samples, and the quantitative data obtained (ng/µL) are summarized in Table 3.

According to the analysis results, the total detected phenolic compound (Total) concentrations in the pollen samples showed a wide distribution between 153.89 ng/µL and 793.94 ng/µL, with an average value of 406.25 ± 162.40 ng/µL. Quercetin was the predominant phenolic compound in the analyzed pollen samples. The amount of quercetin varied between 16.58 ng/µL and 580.37 ng/µL depending on the sample. Quercetin was followed by rutin, t-ferulic acid, caffeic acid, salicylic acid, and t-cinnamic acid, in order of abundance and frequency. Components such as catechin hydrate, naringin, chrysin, and resveratrol could only be specifically detected in samples obtained from certain locations (particularly Marmara and Eastern Anatolia). The highest total phenolic concentration (793.94 ng/µL) was found in sample number 26 from the Central Anatolia Region, while sample number 17 from the Marmara Region was determined to have the highest functional diversity (number of different components).

### 3.4. Correlation Between Phenolic Compounds and Antimicrobial Effect (Spearman Analysis)

**Figure 3:**
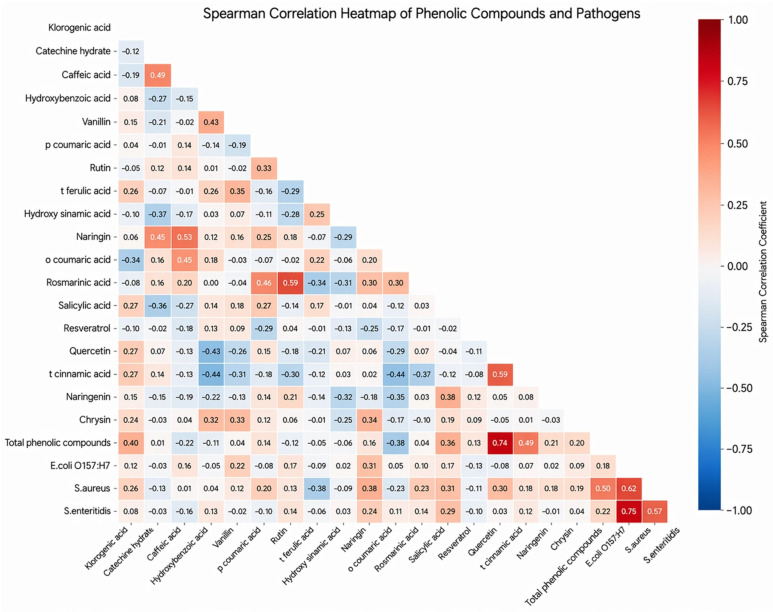
Spearman correlation heatmap illustrating the relationships between phenolic compounds and antimicrobial activity.

Spearman correlation analysis revealed varying degrees of association among phenolic compounds, total phenolic content, and antimicrobial activity. When the relationships between phenolic compounds were examined, moderate-to-strong positive correlations were observed among several compounds. For example, a significant positive relationship was found between catechin hydrate and caffeic acid (r = 0.495, *p* < 0.05), while caffeic acid showed strong positive correlations with both naringin (r = 0.530, *p* < 0.01) and o-coumaric acid (r = 0.450, *p* < 0.01). Similarly, a high level of positive relationship was found between rutin and rosmarinic acid (r = 0.595, *p* < 0.01). However, negative associations were also observed among some compounds; for example, negative correlations were observed between o-coumaric acid and t-cinnamic acid (r = −0.442, *p* < 0.01) and naringenin (r = −0.349, *p* < 0.05). When the relationships between total phenolic compound content and antimicrobial activity were evaluated, it was determined that total phenolic content showed a positive and significant correlation, especially with *S. aureus* (r = 0.503, *p* < 0.01), while the relationship with *E. coli* O157:H7 was weaker and not statistically significant. When the correlations among inhibition responses against the tested microorganisms were examined, strong positive correlations were found between the inhibition zones against *E. coli* O157:H7 and *S. aureus* (r = 0.622, *p* < 0.01), as well as between *E. coli* O157:H7 and *S. Enteritidis* (r = 0.748, *p* < 0.01). This suggests that some pollen samples may exhibit similar antimicrobial activity patterns against different bacterial strains. The results obtained from Spearman correlation analysis are visualized in the heatmap in Figure 3.

Overall, it appears that a complex network of relationships exists among the phenolic compounds, and their effects on antimicrobial activity are too multifaceted to be reduced to a single component. This suggests that the observed antimicrobial effect may be related to the overall effect of the phenolic profile rather than individual compounds.

### 3.5. Presence of Possible Microplastic-Related Chemical Markers in Bee Pollen Samples

Thirty-five bee pollen samples collected from seven different geographical regions were quantitatively analyzed for the presence of plastic-related marker compounds and characteristic pyrolysis products using Pyrolysis–Gas Chromatography–Mass Spectrometry (PY-GC/MS). The microplastic-related compounds and their quantities detected in the pollen samples are presented in Table 4. At least one chemical marker potentially associated with plastic polymers (benzene, phenol, 2,4-dimethyl-1-heptene, 2,4,6,8-tetramethyl-1-undecene, or naphthalene) was detected in 18 of the 35 bee pollen samples (51.4%). Benzene and phenol compounds were the most frequently detected pollutants in the samples.

Benzene was detected in 13 of the samples examined at concentrations ranging from 0.002 mg to 0.006 mg. The highest benzene level (0.006 mg) was recorded in pollen sample number 10 from the Southeastern Anatolia Region.

Phenol was detected at the highest concentration range among the monitored compounds; however, it was reported in ppm because it was measured in the liquid phase. It was detected in a total of 12 samples, showing a wide distribution between 3.192 ppm and 50.934 ppm. The samples showing the highest levels of phenol, a compound that may be associated with certain plastic polymers, were sample number 7 from the Eastern Anatolia Region (50.934 ppm), sample number 10 from the Southeastern Anatolia Region (45.390 ppm), and sample number 24 from the Central Anatolia Region (43.757 ppm), respectively.

Among the microplastic-related chemical markers with an aliphatic hydrocarbon structure, 2,4-dimethyl-1-heptene was measured in equal concentrations (0.007 mg) in only two samples (Sample 3 from Central Anatolia and Sample 6 from Marmara); while the compound 2,4,6,8-tetramethyl-1-undecene was detected in three different samples (Samples 6, 29, and 34) in the range of 0.002 to 0.003 mg. Naphthalene, a polycyclic aromatic hydrocarbon, was detected only in trace amounts in sample number 13 from the Black Sea Region. Since microplastic particles were not directly identified using microscopic or spectroscopic techniques, the detected compounds should be interpreted as polymer-related pyrolysis markers rather than definitive evidence of possible plastic-related contamination.

## 4. Discussion

When examining the microbiological analysis results of the 35 bee pollen samples examined in this study, it was found that, according to commonly cited microbiological quality criteria proposed for commercial bee pollen, TMAB should be below 100,000 CFU/g and yeast–mold counts below 50,000 CFU/g [24,25]. According to our analysis findings, the TMAB load in all samples remained below these recommended limits, ranging from <2.00 to a maximum of 3.23 log CFU/g (Sample 18). Similarly, yeast and mold levels, which can lead to spoilage and mycotoxin production in food, were detected in the range of <2.00 to 4.49 log CFU/g (Table 1). Although yeast and mold counts remained below the proposed tolerance value, their presence should still be considered relevant because mold growth may be associated with potential mycotoxin risk under inappropriate storage conditions [24,26]. Coliform bacteria, a key indicator of fecal contamination and poor hygiene, were not detected in the vast majority of samples (<1.00 log CFU/g) and were observed at very low levels in only a few samples.

The absence of psychrophilic bacteria indicates that cold-tolerant bacterial contamination was not detected under the conditions of this analysis; however, this finding alone is not sufficient to fully assess hygiene during harvesting, drying, and storage [24]. These findings are partly consistent with Demircioğlu et al. [26], who reported the absence of major pathogens in bee pollen samples from Şanlıurfa, although their TMAB and coliform levels were higher than those observed in the present study.

One of the most fundamental factors determining the microbiological quality and shelf life of pollen is the beneficial flora and acidic structure of the product. Previous studies have reported that lactic acid bacteria (LAB), naturally present in bee pollen and which play a leading role in the conversion of bee pollen into bee bread (perga), create a protective barrier against foodborne pathogens and spoilage agents by lowering the pH level of the environment [27,28]. Analysis results showed that beneficial bacteria such as lactobacilli and lactococci were present in some samples, particularly Sample 18, at levels of 3.84 and 4.59 log CFU/g. Previous studies have shown that LAB involved in bee pollen fermentation can produce lactic acid and lower pH, which may contribute to microbial stability by creating less favorable conditions for spoilage and pathogenic microorganisms [2,28].

HPLC analysis of the commercial bee pollen samples examined in this study revealed the highest concentrations of quercetin, rutin, t-ferulic acid, caffeic acid, salicylic acid, and t-cinnamic acid (Table 3). Many studies have shown that bee pollen contains a wide range of bioactive compounds, particularly phenolic acids and flavonoids; bee pollen is a particularly rich natural source of polyphenols and flavonoids (quercetin, rutin, kaempferol, etc.) and phenolic acids (caffeic, ferulic, cinnamic, salicylic acid, etc.) [7,28,29,30]. However, the chemical composition of pollen varies significantly depending on the diversity of plant species visited by bees, geographical origin, flora, and climatic conditions [3,7,25]. The phenolic acid and flavonoid composition identified in our study is consistent with the general bioactive nature of bee pollen and the specific metabolites it contains. Since total phenolic content was not determined using the Folin–Ciocalteu assay, direct comparison with studies reporting total phenolic content as gallic acid equivalents should be interpreted with caution.

In our study, a non-parametric test, Spearman correlation analysis, was applied to determine the relationship between the antimicrobial activity of bee pollen and phenolic compounds (Figure 3). The results show that pathogen inhibition is related to both the total amount of phenolic compounds in the pollen and the specific (qualitative) polyphenol profile. The literature indicates that the antimicrobial activity of bee pollen depends more on the specific composition of phenolic components than on the total phenolic concentration; even extracts with low total phenolic content can show strong inhibition thanks to specific bioactive molecules (phenolic acids and flavonoids); and the antimicrobial effect depends on the specific nature and quality of the bioactive components in the pollen [31,32]. The mechanism of antimicrobial action of these phenolic acids and flavonoids is as follows: The antimicrobial effects of phenolic acids and flavonoids are generally attributed to their ability to interact with bacterial cell envelopes, alter membrane permeability, and interfere with microbial metabolic processes; however, the precise mechanisms may vary depending on the compound and microorganism tested [7,31,33]. A statistically significant and strong positive correlation (r = 0.503, *p* < 0.01) was found between the inhibitory effect on Gram-positive *S. aureus* and the total phenolic content of pollen samples. Naringin concentration also showed a significant positive correlation with *S. aureus* inhibition (r = 0.375, *p* < 0.05) (Figure 3). Previous studies have suggested that phenolic and flavonoid compounds may contribute to the antimicrobial properties of bee products, although the strength of this relationship varies depending on the product type and tested microorganism [34]. Gram-positive bacteria are often reported to be more sensitive to bee pollen extracts than Gram-negative bacteria because Gram-negative bacteria possess an outer lipopolysaccharide membrane and efflux systems; however, this pattern is not universal and may vary depending on pollen type, extract composition and test conditions [31,32,34]. No significant correlation (*p* > 0.05) was found between the inhibition of Gram-negative pathogens *E. coli* O157:H7 and *S. Enteritidis* and either the total phenolic content or individual phenolic components (Figure 3). This may be partly attributed to the complex outer lipopolysaccharide (LPS) membrane structure and efflux pumps of Gram-negative bacteria, which make it difficult for phenolic molecules to enter the cell [31,35]. This weak correlation suggests that inhibition in Gram-negative bacteria is due to minor synergistic interactions of much more specific components, rather than a simple concentration–response (dose–response) relationship.

One of the most striking findings of the correlation analysis is that the amount of t-ferulic acid exhibits a statistically significant negative (inverse) correlation (r = −0.377, *p* <0.05) with *S. aureus* inhibition. These findings are consistent with previous studies suggesting that phenolic and flavonoid compounds contribute to the antimicrobial properties of bee products [34]. These findings suggest that the antimicrobial potential of bee pollen depends more on its specific polyphenol composition than on the total phenolic concentration alone.

Beyond the chemical and microbiological findings of the study, the most innovative result was the detection of volatile and semi-volatile pyrolysis products potentially associated with plastic polymers, such as benzene, phenol, 2,4-dimethyl-1-heptene and 2,4,6,8-tetramethyl-1-undecene in 51.4% of commercial bee pollen samples intended for direct human consumption, via PY-GC/MS analysis (Table 4). Honeybees (*Apis mellifera*) are considered “active bioindicators” for atmospheric and terrestrial microplastics due to the electrostatic charge generated by the hairs on their bodies, their long flight ranges, and their continuous interaction with all environmental matrices such as air, water, soil, and plant flora [9,11,36]. It is known that bees passively trap airborne microfibers and microplastic particles in their bodies, carrying them into the hive and transferring them to pollen, honey, beeswax, and even larvae [9,11,12].

The literature indicates that the source of these plastic-related chemical markers detected in bee products is not solely environmental pollution (urbanization, industrial activities, airborne textile microfibers); it may also be a migration originating from plastic hive frames, synthetic foundation combs, beekeeping clothing, and plastic materials used in the packaging stages of the product during beekeeping practices [9,10,11]. In addition, environmental pollutants carried by wind as a result of agricultural activities, highway traffic, and urbanization are also collected by bees from hive materials or when packaged into the product [11,12]. However, contamination is not limited to environmental exposure alone. Current studies also indicate that microplastics and plasticizers (phthalates, bisphenols, etc.) can migrate from packaging materials to the product during post-harvest processing, transportation, and especially storage in plastic packaging [12,37]. Previous studies have suggested that microplastics and plastic-associated chemicals may adversely affect both bees and human health by altering the gut microbiota of bees, impairing immune function, and increasing susceptibility to pathogens [9,12].

Studies on plastic pollutants in honey and bee products in Türkiye are extremely limited [10]. A review of the literature reveals that in other countries, microplastic pollution in bee pollen is generally detected as physical particles (fibers or fragments), and the most common polymer types are synthetic materials such as polyethylene (PE), polyethylene terephthalate (PET), and polypropylene (PP). In a study conducted in Denmark using bee colonies and bee-related matrices, PET was commonly reported among fibers, while PP and PE were among the detected non-fibrous polymers [14]. In the present study, however, characteristic indicator compounds for PE and PET were not detected, while 2,4-dimethyl-1-heptene, associated with PP, was detected in Samples 3 and 6, and 2,4,6,8-tetramethyl-1-undecene was detected in Samples 6, 29, and 34. In addition, recent studies summarized by Fuente-Ballesteros et al. [11] reported the occurrence of plastic-related compounds in bee pollen, including plasticizers in samples from Spain and bisphenols in samples from both Spain and China. In contrast, the present study detected volatile and semi-volatile pyrolysis products, including phenol, benzene, 2,4-dimethyl-1-heptene, and 2,4,6,8-tetramethyl-1-undecene, which were interpreted as potential polymer-related chemical markers. The detection of phenol and benzene markers (51.4%) may be consistent with multiple possible sources, including environmental deposition, beekeeping materials, processing equipment, or packaging; however, the present screening approach does not allow source apportionment. It should be noted that the PY-GC/MS approach used in this study provides indirect evidence based on polymer-related pyrolysis products and does not constitute direct identification of microplastic particles.

The detection of polymer-related pyrolysis markers in pollen may warrant further investigation from a food safety perspective. Some plastic additives reported in bee products and honey-related matrices, particularly bisphenols and phthalates, are considered toxicologically relevant and may include endocrine-disrupting compounds; however, the specific markers detected in the present study require compound-specific toxicological assessment before risk conclusions can be drawn. Studies on possible plastic-related contamination in honey and bee products from Türkiye remain limited, with current evidence mainly focused on honey [10]. Similarly to other emerging or insufficiently regulated contaminants in bee products, previous Turkish studies on potentially toxic elements have also emphasized the need for establishing regulatory standards and acceptable concentration limits in bee products [38]. The literature also emphasizes that dietary exposure to microplastics through honey consumption may represent an emerging public health concern, as evaluated using parameters such as Estimated Daily Intake (EDI) and Polymeric Hazard Index (PHI) [10]. Indeed, current regulations and international standards do not sufficiently address emerging contaminants in bee products, including microplastic-related chemical markers. Previous studies have also emphasized that specific limits for several contaminants in bee pollen and propolis remain insufficiently defined and that further standardization is needed to ensure product safety [38,39]. These findings highlight the need for further standardization, regulatory monitoring, and evaluation of beekeeping and packaging practices as potential sources of polymer-related contaminants in bee products [10,11]. From a functional food perspective, the rich phenolic composition and antimicrobial activity observed in several bee pollen samples support the potential nutritional and bioactive value of bee pollen. These findings suggest that bee pollen may contribute to the development of value-added functional food products.

## 5. Conclusions

In conclusion, the commercial bee pollen samples examined were found to meet the microbiological criteria reported for safe consumption and did not indicate a major microbiological food safety concern. HPLC analyses revealed that the pollen samples had a phenolic profile rich in quercetin, rutin, and t-ferulic acid; antimicrobial activity appeared to be associated with the synergistic effect of the complex phenolic composition rather than a single phenolic component. However, the detection of polymer-related chemical markers in some samples suggests that bee pollen may serve as a sensitive indicator to environmental pollutants. Based on these findings, it is recommended that more comprehensive studies be conducted on potential microplastic occurrence and polymer-related contaminants and related chemical contaminants in bee products and that safe production and packaging processes be developed.

### Limitations

This study has some limitations. First, the bee pollen samples were commercial products collected from retail outlets; therefore, detailed information on botanical origin, harvesting conditions, processing steps, storage history, and packaging duration could not be fully controlled. Although samples were collected from all seven geographical regions of Türkiye, the findings should be considered exploratory and may not fully represent all commercial bee pollen products available in the country. Second, phenolic compound analysis and PY-GC/MS screening of microplastic-related chemical markers were performed as single instrumental measurements due to technical and cost constraints. Therefore, the results should be interpreted with caution, and future studies should include replicate analyses to improve analytical reliability. Third, PY-GC/MS analysis provides screening-level evidence for selected pyrolysis products potentially associated with plastic polymers, but it does not allow definitive confirmation of physical microplastic particles, polymer identity, or contamination sources without complementary techniques such as microscopy, µFTIR, or µRaman spectroscopy. Finally, the antimicrobial activity was evaluated using an in vitro disc diffusion assay, which may be influenced by the diffusion characteristics, solubility, and molecular weight of bioactive compounds in agar. Therefore, the results should be interpreted as indicative screening data rather than absolute measures of antimicrobial potency. Further studies using purified compounds, MIC/MBC determinations, broader pathogen panels, and in vivo or food-model systems are needed to better clarify the mechanisms and practical relevance of the observed effects.

## Figures and Tables

**Table 1:** Microbial quality results of commercial bee pollen samples (log CFU/g).

Microorganism Group	Number of Samples in Which Growth Was Detected (n = 35)	Mean ± SD	Min–Max Values	95% Confidence Interval (Lower–Upper)
Total Mesophilic Aerobic Bacteria	27 (77.1%)	2.26 ± 0.44	1.69–3.23	2.09–2.43
Coliforms	10 (28.5%)	1.43 ± 0.49	<1.00–2.61	1.13–1.73
Yeast and Molds	26 (74.2%)	2.68 ± 0.69	<2.00–4.49	2.42–2.94
Lactobacilli	9 (25.7%)	2.76 ± 0.54	<2.00–3.84	2.41–3.11
Lactococci	17 (48.5%)	2.81 ± 0.68	<2.00–4.59	2.49–3.13
Psychrophilic Bacteria	0 (0.0%)	Not Detected	<1.00	-

Mean, standard deviation (SD), and 95% Confidence Interval values were calculated based on samples above the detectable limits (positive). N.D.: Not Detected (Below the detection limit in all analyzed samples). < values indicate results below the method detection limit.

**Table 2:** Antimicrobial activity of commercial bee pollen samples against *E. coli* O157:H7, *S. aureus* and *S. Enteritidis*, expressed as inhibition zone diameters (mm, mean ± SD).

Sample Code	Region of Origin	*E. coli* O157:H7	*S. aureus*	*S. Enteritidis*
Sample 1	Eastern Anatolia	-	-	-
Sample 2	Eastern Anatolia	-	-	-
Sample 3	Central Anatolia	-	-	-
Sample 4	Mediterranean	-	-	-
Sample 5	Mediterranean	-	-	-
Sample 6	Marmara	-	-	-
Sample 7	Eastern Anatolia	-	-	-
Sample 8	Southeastern Anatolia	-	-	-
Sample 9	Southeastern Anatolia	10.2 ± 0.2	-	15.3 ± 0.5
Sample 10	Southeastern Anatolia	-	-	-
Sample 11	Southeastern Anatolia	-	-	-
Sample 12	Southeastern Anatolia	-	-	-
Sample 13	Black Sea	-	-	-
Sample 14	Marmara	24.7 ± 0.9	14.7 ± 0.9	14.3 ± 0.5
Sample 15	Marmara	12.0 ± 0.8	20.3 ± 0.5	14.0 ± 0.0
Sample 16	Marmara	30.3 ± 0.5	37.3 ± 1.7	32.7 ± 1.2
Sample 17	Marmara	20.7 ± 1.2	21.7 ± 0.5	13.7 ± 0.9
Sample 18	Eastern Anatolia	-	-	11.3 ± 0.9
Sample 19	Black Sea	8.0 ± 0.0	-	10.3 ± 0.5
Sample 20	Aegean	-	-	14.7 ± 0.9
Sample 21	Aegean	-	-	-
Sample 22	Central Anatolia	-	-	-
Sample 23	Eastern Anatolia	-	-	-
Sample 24	Central Anatolia	31.3 ± 0.5	-	-
Sample 25	Black Sea	-	-	-
Sample 26	Central Anatolia	-	-	-
Sample 27	Central Anatolia	-	-	-
Sample 28	Black Sea	-	9.3 ± 0.5	-
Sample 29	Black Sea	-	-	-
Sample 30	Mediterranean	-	-	-
Sample 31	Mediterranean	-	-	-
Sample 32	Mediterranean	-	-	-
Sample 33	Aegean	-	-	-
Sample 34	Aegean	9.3 ± 0.5	-	12.3 ± 0.5
Sample 35	Aegean	-	-	-
	Gentamicin (positive control)	28.3 ± 0.5	25.7 ± 0.9	25.3 ± 0.5

Values are expressed as mean ± standard deviation (mm) of three independent measurements. Standard deviation values were calculated using population standard deviation (STDEV.P). “-” indicates no detectable inhibition zone.

**Table 3:** Data on the concentrations (ng/µL) of major phenolic components detected by HPLC in bee pollen samples.

Sample Code	Region Where It Is Produced	Clorogenic Acid	Catechin Hydrate	Caffeic Acid	4-Hydroxy Benzoic Acid	Vanillin	p-Coumaric Acid	Rutin	t-Ferulic Acid	Hydroxycinnamic Acid	Naringin	o-Coumaric Acid	Rosmarinic Acid	Salicylic Acid	Resveratrol	Quercetin	t-Cinnamic Acid	Naringenin	Chrysin	Total Phenolic Compounds
Sample 1	Eastern Anatolia	6.71685	-	6.36424	14.61085	51.97602	-	11.40912	89.67052	1.21413	4.97220	7.02915	9.42754	180.73919	27.98089	52.47621	-	6.65831	8.70110	467.90868
Sample 2	Eastern Anatolia	-	-	29.52537	1.70470	4.54980	-	25.82929	43.75804	6.88989	-	2.84316	1.79973	13.33015	9.98759	26.02310	5.85670	5.09451	8.84462	169.90393
Sample 3	Central Anatolia	-	1.14436	32.85906	-	-	10.00021	30.26941	62.00918	2.97263	16.04561	6.78592	7.37086	61.64762	-	54.78109	-	22.74877	2.79850	305.32589
Sample 4	Mediterranean	-	-	33.03884	-	-	14.79098	41.41132	28.74149	7.04195	6.09150	8.47479	9.49682	28.56190	-	60.41180	16.73353	-	-	240.82987
Sample 5	Mediterranean	-	2.39184	16.02563	-	-	7.30455	33.90325	26.99541	1.51407	9.35295	6.48560	8.64586	115.24550	-	67.12309	-	57.83426	-	346.68497
Sample 6	Marmara	-	-	22.48820	-	-	16.37755	37.59018	24.58668	4.25727	7.53168	5.99891	12.13744	6.67298	-	90.09633	43.87577	47.65617	-	310.03859
Sample 7	Eastern Anatolia	7.99436	3.15511	25.24936	-	-	5.60081	9.55110	53.34010	-	17.20877	4.27601	6.72814	3.25941	4.43120	129.44157	127.30375	-	21.08835	402.83713
Sample 8	Southeastern Anatolia	-	3.58068	43.75315	-	-	10.00942	70.94022	41.67098	5.97629	8.26383	4.31622	42.97760	95.48921	20.31360	39.58682	5.03621	10.30205	-	385.19783
Sample 9	Southeastern Anatolia	-	1.38831	30.35523	-	-	-	40.63396	41.25537	1.25775	14.44896	9.98986	16.33736	67.43964	8.09180	68.64823	103.39381	60.56404	8.50208	463.31553
Sample 10	Southeastern Anatolia	2.24916	6.57376	41.00866	-	-	2.16427	42.80831	35.34187	-	13.62806	2.82117	3.42246	2.70432	3.15925	94.52190	90.08707	6.39159	1.13806	345.99567
Sample 11	Southeastern Anatolia	-	9.63613	27.28096	-	6.27847	6.32535	101.72476	27.32125	-	28.44214	3.01710	21.12988	1.91634	2.90392	138.38632	56.14802	3.86536	8.58687	437.73394
Sample 12	Southeastern Anatolia	8.92083	2.22607	17.77350	-	6.16212	6.38049	47.81404	49.39315	14.45582	14.93585	-	11.90480	27.62223	-	167.84528	87.89203	5.13752	7.39239	467.82740
Sample 13	Black Sea	9.92675	-	16.80534	-	-	5.82954	7.51988	46.12852	32.32959	5.54979	-	1.51421	59.24722	-	336.97221	140.74744	40.24500	17.38787	710.26929
Sample 14	Marmara	1.41606	-	21.64213	-	-	5.79303	19.82258	41.44391	31.03929	10.88567	3.22026	3.80062	48.30115	-	298.41822	136.93338	33.60621	-	656.32303
Sample 15	Marmara	-	3.47453	45.32173	-	-	6.70204	145.20693	-	2.21925	42.82715	4.34921	25.95402	4.89530	8.86429	194.39623	59.17535	9.99832	20.13982	560.75447
Sample 16	Marmara	4.84157	-	15.05794	2.94633	17.43439	3.72538	29.32625	26.00689	3.33245	31.69208	2.45523	13.90769	237.43004	-	88.87839	58.75651	3.11584	5.01272	591.71002
Sample 17	Marmara	3.88557	1.76579	29.81542	-	-	7.93668	67.50828	13.84451	3.12733	40.37190	2.65819	10.49750	207.21809	-	161.05286	66.94666	3.18505	27.41782	637.69627
Sample 18	Eastern Anatolia	-	1.33620	-	-	-	-	-	47.44045	13.29220	4.64127	32.19470	4.98763	22.58883	2.03391	336.94063	71.61541	-	-	537.07123
Sample 19	Black Sea	-	2.79852	32.41238	-	10.60866	1.35663	-	42.16558	5.54097	16.02365	5.30629	2.92912	145.62429	-	54.69214	13.22791	-	17.86174	344.55125
Sample 20	Aegean	-	-	-	4.34219	-	1.35342	50.14980	52.26843	9.53246	11.46676	5.80833	9.26540	20.54657	-	49.26510	7.59472	-	-	221.57318
Sample 21	Aegean	-	5.19972	56.85463	3.19525	-	7.38727	51.68271	8.48632	2.63126	83.16329	8.57784	18.34641	-	-	47.67418	5.15817	-	38.35239	328.98939
Sample 22	Central Anatolia	-	-	15.03010	-	-	14.29958	18.36531	26.45882	3.77534	1.08507	-	3.56433	32.61688	-	83.57673	63.20547	33.47074	21.31557	316.76396
Sample 23	Eastern Anatolia	-	-	7.29945	-	-	-	9.53514	2.34577	7.06640	-	1.47721	1.56772	2.62921	6.89559	465.71285	93.92101	-	4.67556	574.07950
Sample 24	Central Anatolia	-	-	61.18446	-	18.75982	-	31.64716	86.14943	12.11896	11.43522	7.27122	3.90749	-	-	26.57144	6.67064	-	-	259.17175
Sample 25	Black Sea	-	1.64512	27.49403	-	-	-	63.18752	19.25215	4.47624	8.07961	9.65274	19.62876	-	-	129.10723	14.42568	-	2.37124	299.32032
Sample 26	Central Anatolia	-	-	-	-	-	23.01914	30.13784	26.92729	5.17436	6.19449	-	27.79876	37.78427	22.30297	580.37982	39.90090	4.55425	-	793.94212
Sample 27	Central Anatolia	6.99295	-	21.30771	-	-	-	37.79237	27.16025	2.68214	5.11322	1.56417	7.20357	18.03899	4.73711	108.99373	31.25052	56.39017	-	322.30386
Sample 28	Black Sea	-	-	18.25518	-	4.46527	2.75477	107.93054	-	4.13473	10.86953	-	11.32040	32.83357	4.59266	158.98838	41.35176	148.62476	2.92867	546.57092
Sample 29	Black Sea	-	-	-	-	-	-	9.61990	1.52354	4.27671	10.73467	-	1.16400	50.49703	8.16249	36.19492	161.83363	24.41352	6.60680	306.35928
Sample 30	Mediterranean	-	5.55436	23.69091	-	-	-	16.41820	44.56470	9.19156	8.91516	1.59824	1.06324	2.97821	1.65712	127.58295	218.35022	-	-	453.29246
Sample 31	Mediterranean	-	2.08278	61.39213	-	-	4.01949	-	3.87920	7.11198	18.65573	13.05116	38.44715	18.23804	-	141.17711	6.35618	-	-	304.39263
Sample 32	Mediterranean	-	7.30882	49.98282	-	-	1.16840	-	27.01776	4.76883	15.51657	6.16320	6.71894	2.58460	4.93217	135.02462	125.83298	-	-	378.28881
Sample 33	Aegean	-	-	26.45307	2.95118	8.75019	1.97159	13.35903	85.13021	40.75928	23.88634	5.77645	1.46379	42.74766	11.35800	66.05445	5.66263	2.50213	57.23487	390.85207
Sample 34	Aegean	-	4.22355	14.77397	-	-	-	50.35691	24.38213	6.82456	4.93716	2.71791	8.46574	-	9.59213	16.58601	6.06957	8.76283	-	153.89127
Sample 35	Aegean	-	13.27233	34.63019	-	-	-	-	37.77576	2.36608	15.10924	2.77939	3.51731	9.61942	-	211.22051	176.69724	49.78759	-	556.77506

Total phenolic content was calculated as the sum of individually identified phenolic compounds.

**Table 4:** Chemical markers potentially associated with microplastic polymers detected in bee pollen samples by PY-GC/MS.

Sample Code	Region Where It Is Produced	2,4-Dimethyl-1-heptene (mg)	Benzene (mg)	2,4,6,8-Tetramethyl-1-undecene (mg)	Phenol (ppm)	Naphthalene (mg)
Sample 1	Eastern Anatolia	-	-	-	-	-
Sample 2	Eastern Anatolia	-	-	-	-	-
Sample 3	Central Anatolia	0.007	0.004	-	-	-
Sample 4	Mediterranean	-	-	-	-	-
Sample 5	Mediterranean	-	0.002	-	-	-
Sample 6	Marmara	0.007	-	0.002	-	-
Sample 7	Eastern Anatolia	-	0.003	-	50.934	-
Sample 8	Southeastern Anatolia	-	-	-	-	-
Sample 9	Southeastern Anatolia	-	-	-	-	-
Sample 10	Southeastern Anatolia	-	0.006	-	45.390	-
Sample 11	Southeastern Anatolia	-	-	-	-	-
Sample 12	Southeastern Anatolia	-	-	-	-	-
Sample 13	Black Sea	-	0.004	-	-	0.000
Sample 14	Marmara	-	0.002	-	31.764	-
Sample 15	Marmara	-	-	-	-	-
Sample 16	Marmara	-	0.002	-	27.712	-
Sample 17	Marmara	-	-	-	-	-
Sample 18	Eastern Anatolia	-	0.002	-	12.827	-
Sample 19	Black Sea	-	-	-	-	-
Sample 20	Aegean	-	0.002	-	-	-
Sample 21	Aegean	-	0.005	-	8.218	-
Sample 22	Central Anatolia	-	-	-	-	-
Sample 23	Eastern Anatolia	-	-	-	-	-
Sample 24	Central Anatolia	-	0.002	-	43.757	-
Sample 25	Black Sea	-	-	-	-	-
Sample 26	Central Anatolia	-	-	-	3.192	-
Sample 27	Central Anatolia	-	-	-	-	-
Sample 28	Black Sea	-	-	-	-	-
Sample 29	Black Sea	-	0.002	0.003	10.328	-
Sample 30	Mediterranean	-	0.002	-	-	-
Sample 31	Mediterranean	-	-	-	9.073	-
Sample 32	Mediterranean	-	-	-	-	-
Sample 33	Aegean	-	-	-	6.832	-
Sample 34	Aegean	-	-	0.002	15.126	-
Sample 35	Aegean	-	-	-	-	-

## Data Availability

The original contributions presented in this study are included in the article. Further inquiries can be directed to the corresponding author.
